# Stereotactic total ablative radiotherapy with MR-LINAC for synchronous oligometastatic prostate cancer

**DOI:** 10.3389/fonc.2025.1607610

**Published:** 2025-07-03

**Authors:** Darren MC. Poon, Jing Yuan, Oi Lei Wong, Bin Yang, Sin Ting Chiu, Kin Yin Cheung, George Chiu, Siu Ki Yu

**Affiliations:** ^1^ Comprehensive Oncology Centre, Hong Kong Sanatorium & Hospital, Hong Kong, Hong Kong SAR, China; ^2^ Research Department, Hong Kong Sanatorium & Hospital, Hong Kong, Hong Kong SAR, China; ^3^ Medical Physics Department, Hong Kong Sanatorium & Hospital, Hong Kong, Hong Kong SAR, China; ^4^ Department of Radiotherapy, Hong Kong Sanatorium & Hospital, Hong Kong, Hong Kong SAR, China

**Keywords:** oligometastatic prostate cancer (OMPC), stereotactic total ablative radiotherapy (STAR), magnetic resonance-guided radiotherapy (MRgRT), toxicity, progression-free survival (PFS)

## Abstract

**Objectives:**

To prospectively investigate the feasibility, toxicity, and preliminary clinical outcomes of magnetic resonance (MR)-guided stereotactic total ablative radiotherapy (MRgSTAR) for simultaneous treatment of the prostate and pelvic bone metastases in patients with synchronous oligometastatic prostate cancer (OMPC).

**Methods:**

This study included patients with histologically confirmed synchronous OMPC, defined as ≤ 5 lymph node or pelvic bone metastases identified via prostate-specific membrane antigen positron emission tomography (PSMA-PET). Real-time adaptive MRgSTAR was delivered using a 1.5T MR-integrated linear accelerator (MR-LINAC) in five fractions, administered twice weekly, targeting the prostate (33.5–40 Gy) and nodal/bone metastases (36.5–40 Gy) simultaneously. Androgen-deprivation therapy (ADT) was initiated prior to MRgSTAR, with the addition of androgen receptor pathway inhibitor (ARPI) at the physician’s discretion. Adverse events (AEs) were assessed using the Common Terminology Criteria for AEs v5.0, and tumor response was evaluated per the Response Evaluation Criteria in Solid Tumors v1.1. Progression-free survival (PFS) and overall survival (OS) were estimated using the Kaplan–Meier method, with log-rank tests used to explore clinical factors associated with survival outcomes.

**Results:**

Forty-three patients underwent MRgSTAR, with a median follow-up of 36.5 months (range: 15.4–57.6 months). ADT combined with ARPI therapy was administered in 22 patients (52%). All patients completed the five-fraction regimen. Biochemical progression occurred in three patients, of whom two had out-of-field metastases and one had local progression as per follow-up PSMA-PET. The estimated 3-year OS and PFS rates were 100% and 95.2% (95% confidence interval: 89.0%–100%), respectively. No clinical factors, including ARPI use, significantly correlated with survival outcomes. No radiotherapy-related AEs of grade ≥ 3 were observed.

**Conclusion:**

MRgSTAR demonstrates promising early survival outcomes and a favorable toxicity profile in synchronous OMPC, warranting further investigation to confirm its therapeutic role.

## 1 Introduction

Prostate cancer remains a significant global health burden, particularly in its metastatic forms (1). Synchronous oligometastatic prostate cancer (OMPC), defined as five or fewer metastatic lesions (typically lymph nodes or bones) at initial diagnosis, represents an intermediate state between localized and widely metastatic disease, offering an opportunity for aggressive local therapy (2). The advent of advanced imaging tools, such as prostate-specific membrane antigen positron emission tomography (PSMA-PET), has enhanced the detection of OMPC (3). Current standard care includes systemic therapies such as androgen-deprivation therapy (ADT) combined with androgen receptor pathway inhibitors (ARPIs), with or without docetaxel; however, disease progression remains a challenge (4–8). Robust evidence supports prostate-directed radiotherapy (PDRT) in hormone-sensitive OMPC, with trials such as HORRAD (9, 10) and STAMPEDE (11, 12) demonstrating survival benefits for PDRT combined with ADT in low-burden disease.

Despite these advances, untreated metastatic lesions often contribute to disease progression. Stereotactic body radiotherapy (SBRT) or stereotactic ablative radiotherapy (SABR) has gained recognition as an effective treatment for oligometastatic disease, delivering high doses of radiation to metastatic lesions with precision while minimizing damage to surrounding tissues (13). Simultaneous stereotactic total ablative radiotherapy (STAR) targeting both the prostate and metastases may further improve outcomes, yet data specific to synchronous OMPC are limited. A key challenge with STAR is the potential for increased treatment-related toxicity when concurrently irradiating multiple targets. Magnetic resonance-guided STAR (MRgSTAR), utilizing the superior soft-tissue contrast and real-time adaptation capabilities of MR-integrated linear accelerators (MR-LINACs), offers a promising approach to address these issues (14). This technology enables precise targeting and toxicity reduction across multiple treatment sites (15–18).

This study assessed the feasibility, toxicity, and preliminary clinical outcomes of MRgSTAR for synchronous OMPC, targeting both the prostate and pelvic metastatic lesions using a 1.5T MR-LINAC. By addressing unmet needs in local control, toxicity minimization, and survival improvement, this research aims to contribute to the evolving management of OMPC and provide a basis for future large-scale studies.

## 2 Materials and methods

### 2.1 Patient selection

This prospective study was approved by our institutional research ethics committee (REC-2021-28). The informed consent was obtained from all study participants. This study enrolled patients with histologically confirmed prostate cancer scheduled for treatment with a 1.5T MR-LINAC. Inclusion criteria comprised age ≥ 18 years, no prior malignancy, no contraindications to magnetic resonance imaging (MRI), and synchronous OMPC (≤ 5 lymph node or bone metastases) confirmed by PSMA-PET within 6 months of initial prostate cancer diagnosis and prior to systemic therapy. Patients with prior prostate surgery or radiotherapy were excluded, though prior systemic therapy before MRgSTAR was permitted. Exclusion criteria included unwillingness to provide consent, MRI contraindications, absence of pre-MRgSTAR PSMA-PET, > 5 metastatic lesions or visceral metastases, oligo-recurrent/progressive/resistant prostate cancer, metastatic castration-resistant prostate cancer (mCRPC), prior prostate surgery or irradiation, and follow-up < 3 months.

### 2.2 Simulation and planning

Fiducial markers were deemed unnecessary under MRI guidance, and rectal spacer implantation was optional. Patients underwent same-day computed tomography (CT) and MRI simulation scans in the treatment position, with a full bladder (130–160 mL, confirmed by ultrasound) and an empty rectum facilitated by a 50–90 mL saline-inflated rectal balloon (QLARD, Miami, FL, USA). MRI simulation utilized a 1.5T scanner with a three-dimensional T2-weighted turbo spin echo (3D-T2W-TSE) sequence, aligned with daily MR-LINAC imaging parameters.

**Figure 1:**
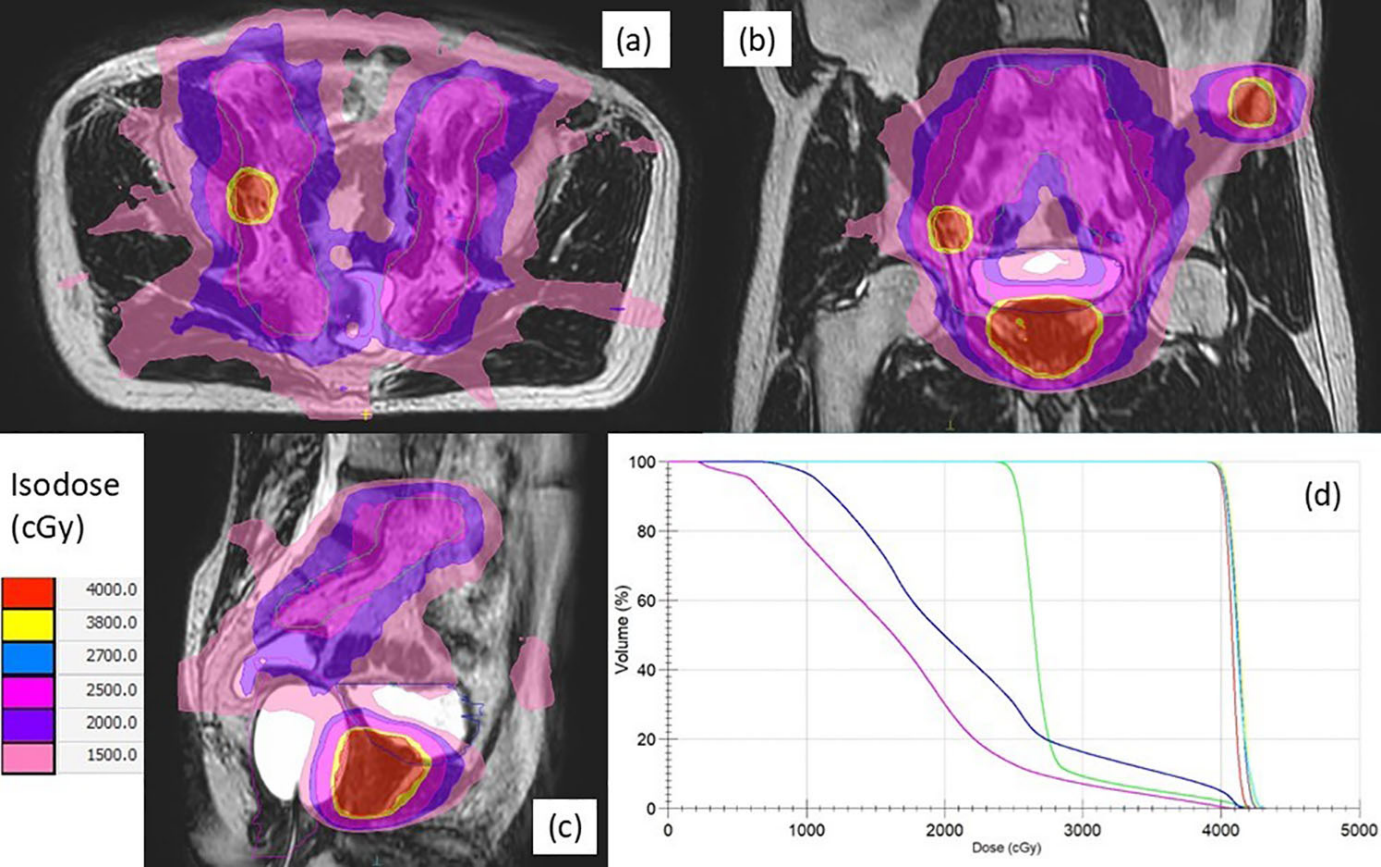
Axial **(a)**, coronal **(b)**, and sagittal **(c)** views of a typical plan of magnetic resonance-guided stereotactic total ablative radiotherapy for a synchronous oligometastatic prostate cancer patient with both nodal and bone metastases, along with dose-volume histograms **(d)**. The planning target volumes for the prostate (red), lymph node (yellow), bone (cyan), lymphatics (green), bladder (blue), and rectum (magenta) are indicated by solid lines, respectively. Isodose levels are illustrated using the indicated colors.

Treatment plans were developed using Monaco v5.40 (Elekta, Stockholm, Sweden) with a Monte Carlo algorithm accounting for magnetic field effects. MRgSTAR was delivered in five fractions (2–3 per week), administering 7.25–8 Gy/fraction to the prostate clinical target volume (CTV), 6.5–8 Gy/fraction to PSMA-PET-identified metastases, and 8.5 Gy/fraction to the MRI-visible dominant intraprostatic lesion (DIL). Radiation to pelvic lymphatics (optional, 5 Gy/fraction) included the obturator, external iliac, proximal internal iliac, presacral, and common iliac nodes up to L4–L5. Organs-at-risk (OARs), such as the rectum, bladder, and femoral heads, were contoured per institutional guidelines. CTV-to-planning target volume (PTV) margins were 5 mm (3 mm posteriorly) for the prostate, 3–5 mm for the DIL and nodal metastases, 5–10 mm for bone metastases, and 5 mm for lymphatics (**Figure 1**). Dosimetric criteria are detailed in **Supplementary Table S1**. Extra-pelvic metastases exceeding the MR-LINAC field (22 cm superior-inferior) were separately treated with SBRT using alternative platforms (e.g. CyberKnife).

### 2.3 Treatment delivery and adaptation

Bowel and bladder preparation mirrored simulation conditions, maintaining bladder volume within ± 20% of reference. Daily on-board MRI scans (3D-T2W-TSE) using the MR-LINAC captured daily anatomical data to inform the appropriate online adaptation strategy. Based on these MRI images, either an adapt-to-position (ATP) or adapt-to-shape (ATS) approach was implemented based on institutional criteria and the expertise of the attending oncologist. The ATP workflow, which re-optimized plans based on isocenter shifts without re-contouring of the target or OARs, was generally prioritized to optimize workflow efficiency. ATS involved manual contour refinement by oncologists, followed by plan re-optimization. Priority was given to achieving optimal target coverage while adhering to dose constraints for OARs. A second MRI verified positioning, with additional ATP if needed. No motion monitoring was employed during beam delivery.

### 2.4 Systemic therapy

Systemic therapy, including continuous ADT (luteinizing hormone-releasing hormone agonists/antagonists or orchiectomy), was administered prior to MRgSTAR. The addition of ARPIs (e.g. enzalutamide) was optional and at the oncologist’s discretion. Docetaxel was not considered in our cohort as its benefit in low volume metastatic prostate cancer remains uncertain.

### 2.5 Follow-up and outcomes

Follow-up was conducted at 1, 3, and 6 months post-MRgSTAR, and thereafter every 6 months. Prostate-specific antigen (PSA) levels were measured at each visit. PSMA-PET was performed in cases of persistent PSA progression or emergence of symptoms. Adverse events (AEs) were graded per the Common Terminology Criteria for Adverse Events v5.0, and tumor response assessed via the Response Evaluation Criteria in Solid Tumors v1.1. Primary endpoints were radiographic progression-free survival (rPFS, i.e. the time from OMPC diagnosis to radiographic progression or death) and biochemical progression-free survival (bPFS, i.e. the time to two consecutive PSA increases ≥ 50% above nadir or death). Secondary endpoints included progression-free survival (PFS, i.e. radiographic/biochemical progression or death), overall survival (OS), and grade ≥ 2 AEs. Patients without events were censored at the last follow-up.

### 2.6 Statistical analysis

Analyses were conducted using RStudio v1.2 (Boston, MA, USA). Continuous data were reported as medians (ranges), and categorical data as percentages. The follow-up duration was calculated from OMPC diagnosis to progression or the last visit. Survival endpoints were estimated using the inverse Kaplan–Meier method, with differences assessed via log-rank tests across variables (e.g. T stage, Gleason score, and ARPI use). Significance was set at P value < 0.05, adjusted with the Bonferroni correction.

## 3 Results

Between June 2020 and December 2023, 67 patients with PSMA-PET-diagnosed metastatic prostate cancer underwent 1.5T MR-guided SBRT at our institution. After excluding 24 patients with > 5 metastases, oligo-recurrent/progressive/resistant prostate cancer, prior prostatectomy, mCRPC, or follow-up < 3 months, 43 patients with synchronous OMPC were included (**Table 1**).

**Table 1:** Patient characteristics at baseline.

Patient Characteristics	All patients (N = 43)
Median age (range) at MRgRT, years	66.9 (45.6–93.5)
Median prostate CTV (range), cc	39.0 (15.8–106.7)
Median metastatic node GTV (range), cc	0.8 (0.2–8.1)
Median metastatic bone GTV (range), cc	1.7 (0.3–12.4)
Median pre-MRgSBRT PSA level (range), ng/mL	34.0 (4.3–367.0)
Histological Gleason score, n (%)	
3 + 3	3 (7.0)
3 + 4	3 (7.0)
3 + 5	2 (4.6)
4 + 3	7 (16.3)
4 + 4	4 (9.3)
4 + 5 or 5 + 4	20 (46.5)
5 + 5	3 (7.0)
NA	1 (2.3)
Clinical T stage, n (%)	
T2a	1 (2.3)
T2b	0
T2c	10 (23.3)
T3a	5 (11.6)
T3b	19 (44.2)
T4	5 (11.6)
NA	3 (7.0)
Clinical N stage, n (%)	
N0	11 (25.6)
N1	32 (74.4)
Clinical M stage, n (%)	
M0	12 (27.9)
M1	31 (72.1)
Patients with irradiated oligometastases by MRgSTAR, n (%)	
Single metastatic target	15 (34.9)
Two metastatic targets	7 (16.3)
Three metastatic targets	8 (18.6)
Four metastatic targets	6 (14.0)
Five metastatic targets	7 (16.3)
Target irradiated oligometastases, number of lesions	
MRgSTAR on MR-LINAC	
Intra-pelvic metastatic lymph nodes	88
Intra-pelvic metastatic bones	24
Non-MRgSTAR on other treatment machines	
Distant (extra-pelvic) metastatic lymph nodes	6
Distant (extra-pelvic) metastatic bones	21
Patients with systemic therapy, n (%)	
ADT only	21 (48.8)
ADT + ARPI	21 (48.8)
ADT + docetaxel	0
ADT + ARPI + docetaxel	1 (2.4)

ADT, androgen deprivation therapy; ARPI, androgen receptor pathway inhibitor; CTV, clinical target volume; GTV, gross tumor volume; MRgRT, magnetic resonance-guided radiotherapy; MRgSBRT, magnetic resonance-guided stereotactic body radiotherapy; MRgSTAR, magnetic resonance-guided stereotactic total ablative radiotherapy; NA, not available; PSA, prostate-specific antigen.

All patients completed MRgSTAR, targeting 43 prostates, 88 lymph nodes, and 24 bone lesions. Most patients (65.1%; 28/43) had ≥ 2 metastatic targets, with 16.3% (7/43) having five. ATP and ATS were used in 168 (78%) and 47 (22%) fractions, respectively, with a median fraction duration of 60 minutes (range: 27–220 minutes). Extra-pelvic metastases in 12 patients were separately treated with SBRT prior to MRgSTAR. Among these 12 patients, extra-pelvic metastatic nodes (n = 6) and extra-pelvic bone metastases (n = 21) were irradiated using a LINAC (n = 7), helical tomotherapy (n = 1), and CyberKnife (n = 7). ADT combined with ARPI therapy was administered in 22 patients (52%).

**Figure 2:**
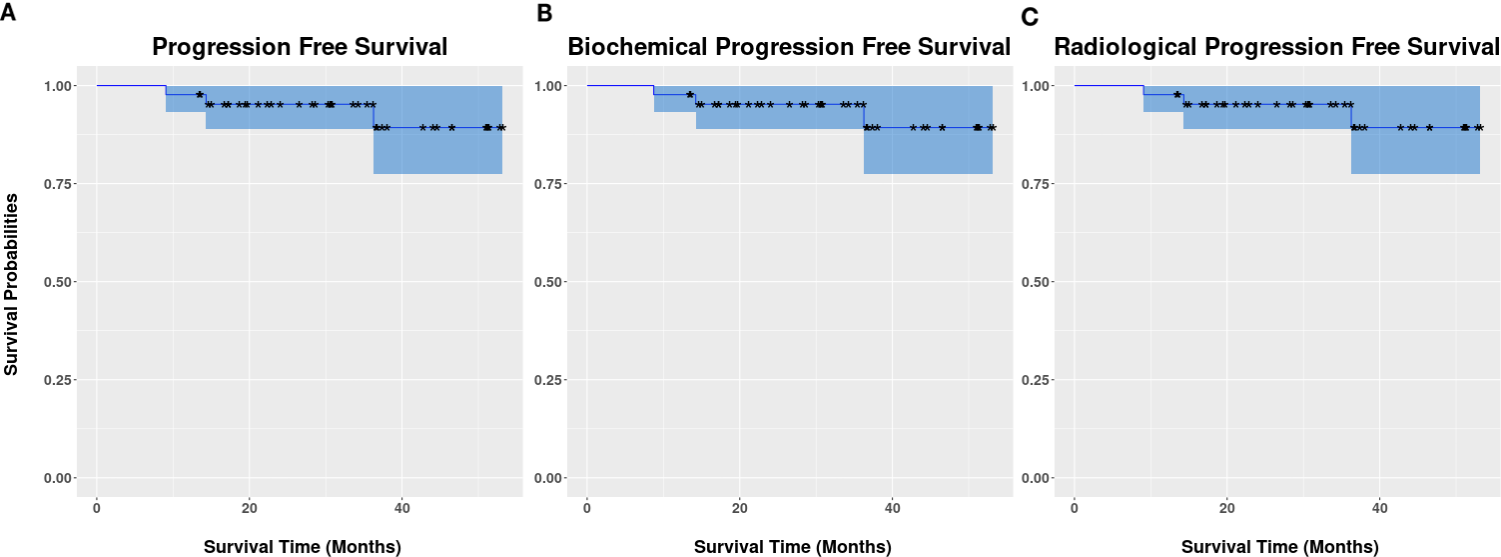
Kaplan–Meier survival curves of progression-free survival **(A)**, biochemical progression-free survival **(B)**, and radiographic progression-free survival **(C)**.

The median follow-up duration was 36.5 months (range: 15.4–57.6 months). Three patients had biochemical progression at 9, 14, and 36 months post-MRgSTAR, with two having out-of-field progression and one having local progression as confirmed using PSMA-PET at 10, 14, and 36 months. In 13 patients who were assessed via follow-up PSMA-PET, no local persistence/progression in treated lesions was noted. All patients remained alive at the last follow-up, yielding a 3-year OS rate of 100%. The rates of bPFS, rPFS, and PFS were identically 95.2% (95% CI: 89.0%–100%) (**Figure 2**).

Regarding MRgSTAR-related genitourinary (GU) and gastrointestinal (GI) toxicities, 9.3% (4/43) of patients experienced four acute grade 2 GU adverse events (AEs) within 3 months, all of which subsequently resolved. No subacute or late GU AEs of grade ≥ 2 were reported. Two patients (2/43, 4.7%) experienced subacute grade 2 GI AEs (rectal hemorrhage). One patient with an acute grade 2 GU AE also exhibited an acute grade 2 elevation in alanine aminotransferase, potentially unrelated to MRgSTAR. Additionally, 9.3% (4/43) of patients experienced late grade 2 non-GI/GU AEs. One patient (2.3%) developed subacute grade 3 neutropenia 4 months post-MRgSTAR, likely associated with ARPI use. **Table 2** lists the details of AEs.

**Table 2:** Incidences of clinician-reported adverse events (grade [G] ≥ 2) based on the Common Terminology Criteria for Adverse Events (CTCAE) v5.0.

Follow-up phase		Baseline		Acute		Subacute		Late	
Toxicity grade (CTCAE v5.0)		G2	G3	G2	G3	G2	G3	G2	G3
GU Toxicity	Cystitis noninfective (dysuria)	0	0	2	0	0	0	0	0
	Urinary hesitancy	0	0	1	0	0	0	0	0
	Urinary frequency	0	0	1	0	0	0	0	0
	Cystitis noninfective (nocturia)	0	0	1	0	0	0	0	0
GI Toxicity	Rectal hemorrhage	0	0	0	0	2	0	0	0
Other Toxicity	ALT increase	0	0	1	0	0	0	0	0
	Febrile neutropenia	0	0	0	0	0	1	0	0
	Fatigue	0	0	0	0	1	0	0	0
	Neuropathy	0	0	0	0	0	0	1	0
	Edema (limbs)	0	0	0	0	0	0	2	0
	Paronychia	0	0	0	0	0	0	1	0

ALT, alanine aminotransferase; GI, gastrointestinal; GU, genitourinary.

The log-rank test revealed no significant differences in rates of rPFS, bPFS, PFS, and OS, or toxicity across stratification factors (including clinical T stage, Gleason score, pre-radiotherapy PSA level, number of metastatic lesions, presence of bone or distant metastasis, ARPI use, and 1-month post-radiotherapy PSA level; **Supplementary Table A2**). However, these results should be interpreted with caution due to a limited number of events and a small sample size.

## 4 Discussion

This study represents the first prospective evaluation of MRgSTAR for the simultaneous treatment of the prostate and pelvic oligometastatic lesions in patients with synchronous OMPC. Our results demonstrated that MRgSTAR offers low toxicity and promising early survival outcomes, potentially serving as a feasible and novel therapeutic strategy for synchronous OMPC.

Standard management of synchronous OMPC typically involves PDRT combined with ADT, as supported by the STAMPEDE (12) and HORRAD (9, 10) trials, which reported the survival benefits of the combination therapy over ADT alone. However, these trials did not address oligometastatic lesions, potentially leaving reservoirs for disease progression. In contrast, MRgSTAR targets both the prostate and metastases, offering a comprehensive approach to local control. With a median follow-up of 36.5 months, our cohort achieved a 3-year OS rate of 100% and PFS rate of 95.2% (95% CI: 89.0–100%). These outcomes appeared to be more favorable than data reported in STAMPEDE (3-year OS rate in low-burden metastatic patients: 81%) and HORRAD (median OS: ~45 months) (9, 10, 12). The enhanced survival outcomes in our study may be attributed to the ability of MRgSTAR (augmented by precise PSMA-PET staging) to aggressively target all detectable disease sites. However, direct comparisons are confounded by differences in imaging modalities (PSMA-PET versus conventional imaging) and systemic therapy regimens (ADT ± ARPI therapy versus ADT alone).

The role of metastasis-directed radiotherapy (MDRT) is well-established in metachronous OMPC, with trials such as ORIOLE (19) and STOMP (20) demonstrating the benefits of SABR in prolonging PFS and delaying initiation of systemic therapy. The application of MDRT in synchronous OMPC, however, remains less defined (21, 22). Siva et al. (23) reported that total metastatic ablation improved OS and PFS in patients with oligometastatic disease, including a subset with synchronous prostate cancer, supporting a broader use of MDRT. Similarly, the EXTEND study (24) demonstrated that MDRT, combined with intermittent hormone therapy, delayed time to progression in patients with OMPC, reinforcing the potential role of MDRT in altering the disease trajectory. Our study extended the MDRT paradigm to synchronous OMPC and suggested that simultaneous treatment of all lesions using MRgSTAR may similarly disrupt disease progression by eliminating metastatic subclones. Given their distinct biological and clinical profiles, synchronous and metachronous OMPC may require tailored therapeutic approaches. Our findings on MRgSTAR targeting all lesions implied the potential benefits of MDRT for patients with synchronous OMPC, shedding some light on the evolving therapeutic framework for this patient population.

Systemic therapy intensification with ARPI therapy alongside ADT is a cornerstone of metastatic castration-sensitive prostate cancer (mCSPC) management, with trials such as TITAN reporting a 3-year PFS rate of ~80% in low-volume disease (6). The better outcome in our study (3-year PFS rate: 95.2%) may be attributed to the strength of MRgSTAR to achieve precise local control. Notably, we observed that ARPI use and survival outcomes had no significant association, but a small sample size (n = 43) and a low number of progression events (n = 3) were the major limitations. Given the demonstrated efficacy of ARPIs in low-volume mCSPC and the capability of MRgSTAR to target macrometastases, the combination of these treatment modalities, along with ADT, is worth considering. Nonetheless, the effectiveness of MRgSTAR in local control may reduce the necessity for systemic therapy intensification or indicate a potential for treatment de-escalation in certain patients. This hypothesis should be validated in further trials.

MRgSTAR demonstrated a favorable toxicity profile in our cohort of patients with synchronous OMPC, of whom 9.3% and 4.7% having acute grade 2 GU toxicity and subacute grade 2 GI toxicity, respectively. No late grade ≥ 2 GU or GI toxicities were reported. These findings were more favorable than outcomes reported in studies utilizing conventional non-MRI–guided radiotherapy techniques. Montero et al. (25), Reverberi et al. (26), Imber et al. (27), and Deantoni et al. (28) conducted studies using conventional radiotherapy, SBRT, or intensity-modulated radiation therapy on 25–50 patients with follow-up periods ranging from 18–30 months. Of the participants across these studies, 15–20% had acute grade 2 GU toxicity, 5% had acute grade 3 GU toxicity [only reported in Reverberi et al. (26)], 8–15% had late grade 2 GU toxicity, 10–15% had acute grade 2 GI toxicity, and 4–8% had late grade 2 GI toxicity. Ingrosso et al. (29) reported that patients treated with volumetric image-guided moderately hypofractionated radiotherapy with daily cone-beam CT for localized prostate cancer had very low rates of late grade ≥ 3 GU (1.6%) and GI toxicities (0.9%). The lower rates of toxicities reported in our study may be associated with MRI guidance that facilitated precise radiation to target tumors and reduced irradiation to normal structures. These outcomes suggest that, with a favorable toxicity profile, MRgSTAR may serve as a promising approach to total ablative radiotherapy in OMPC.

Despite demonstrating the strengths of MRgSTAR, this study had several limitations. First, the small sample size and short follow-up duration precluded definitive conclusions about long-term efficacy and late toxicities of MRgSTAR. Second, due to the absence of a concurrent control group, we could only compare our data with historical cohorts in literature, restricting the generalizability of our findings. Third, the use of PSMA-PET for staging, although highly sensitive, may have introduced selection bias, potentially compromising the representativeness of the study cohort. Fourth, our outcomes may have been confounded by the heterogeneity of the patient cohort due to the inclusion of a subgroup with ARPI use, whereas we analyzed the outcomes stratified by treatment type to address this caveat, with the aim of clarifying the specific contributions of MRgSTAR. Moreover, in real-life clinical practice, the adoption of MRgSTAR remains narrow due to barriers including the limited availability of MR-LINACs and technical challenges such as extended treatment durations.

Several ongoing studies are investigating the combination of PDRT and MDRT for the treatment of synchronous OMPC. The CORE trial (NCT02759783) is a randomized study that compares standard systemic therapy alone versus systemic therapy combined with SABR in patients with prostate cancer and 1–3 oligometastatic lesions. Additionally, the STAMPEDE2 trial (NCT06320067) in the United Kingdom is examining the efficacy of intensified local and systemic therapies, including MDRT, in men with *de novo* metastatic prostate cancer and those with synchronous OMPC. These studies are designed to provide high-level evidence to inform the integration of MDRT into the treatment paradigm for OMPC, which is expected to validate or refine our study findings.

## 5 Conclusions

The highly promising results of our study indicate a substantial potential for integrating MRgSTAR into the management of OMPC. The outcomes of MRgSTAR should be further validated in larger trials. To expand its adoption, access to MR-LINACs should be enhanced. Despite these limitations, our research underscores the possibility of MRgSTAR as a more effective and precise radiotherapy method to improve clinical outcomes in patients with OMPC.

## Data Availability

The original contributions presented in the study are included in the article/**Supplementary Material**. Further inquiries can be directed to the corresponding author.
