# Supplementary material for: Stereotactic total ablative radiotherapy with MR-LINAC for synchronous oligometastatic prostate cancer
Source: Front Oncol. 2025 Jul 3;15:1607610. doi: 10.3389/fonc.2025.1607610 (PMC12268278; doi:10.3389/fonc.2025.1607610)
Supplement: Supplementary file 1 [file Table1.docx]

Table S1. Dosimetric criteria of target organs and organs-at-risk in magnetic resonance-guided stereotactic total ablative radiotherapy treatment plans.

| Structure | Planning objectives |
| --- | --- |
| *PTV_Primary* | V105% < 0.03 cc |
|  | V100% > 95% |
| *GTV_Boost* | V110% < 1 cc  V105% > 95% |
| *PTV_LN* | V90–107% < 1 cc  V81.25–100% > 95% |
| *PTV_Bone* | V90–107% < 1 cc  V81.25–100% > 95% |
| *PTV_L* | V27 Gy < 20%  V25 Gy > 95% |
| *Rectum* | V104% < 1 cc |
|  | V95% < 3 cc |
|  | V90% < 10% |
|  | V80% < 20% |
|  | V50% < 50% |
| *Bladder* | V104% < 1 cc |
|  | V92.5% < 5 cc |
|  | V90% < 10% |
|  | V50% < 50% |
|  | V45% < 60% |
| *Femoral Head* | V20 Gy < 10 cc |
|  | D_max_ < 30 Gy |
| *Penile Bulb* | D2% < 28.5 Gy |
|  | V20 Gy < 3 cc |
|  | D_max_ < 100% |
|  | D_mean_ < 40% |
| *Bowel Space_nPTV* | V25 Gy < 195 cc |
|  | V12.5 Gy < 830 cc |
| *Cauda Equina Body* | D_max_ < 32 Gy |
| *Sacral Nerve Root* | D_max_ < 32 Gy |

GTV, gross tumor volume; L, lymphatics; LN, lymph node; PTV, planning target volume.

Table S2. The effect of stratification factors on clinical outcomes.

| **Variable** | **Patient no.**  **(N = 43)** | ***p*-value** | | |
| --- | --- | --- | --- | --- |
|  |  | **bPFS*** | **rPFS*** | **≥ grade 2 toxicities^†^** |
| T stage^‡^ | | | | |
| T2  T3  T4 | 11  24  5 | 0.3 | 0.3 | 0.6 |
| International Society of Urological Pathology grade^¶^ | | | | |
| > 3  ≤ 3 | 27  15 | 0.8 | 0.8 | 0.2 |
| Number of metastatic lesions | | | | |
| > 3  ≤ 3 | 13  30 | 0.4 | 0.4 | 0.5 |
| Presence of bone metastasis | | | | |
| Yes  No | 20  23 | 0.5 | 0.5 | 0.7 |
| Use of androgen receptor pathway inhibitors | | | | |
| Yes  No | 22  21 | 0.06 | 0.06 | 0.6 |
| 1-month post-radiotherapy prostate-specific antigen level (ng/mL) | | | | |
| ≤ 0.1  > 0.1 | 13  30 | 0.3 | 0.3 | 0.5 |

*Results from the log-rank test.

^†^Results from the logistic regression analysis.

^‡^Information on the T stage was not available in three patients.

^¶^Information on the Gleason score was not available in one patient.
